# Clinical Efficacy and Safety Assessment of Specific-Mode Electroacupuncture Stimulation Combined With Paclitaxel for Recurrent Malignant Gliomas: Study Protocol for a Single-Arm Trial

**DOI:** 10.2196/84593

**Published:** 2026-01-29

**Authors:** Zhaoxing Jia, Tianxiang Jiang, Yiqing Zhang, Qianyue Chen, Zhong Di, Qi Yuan, Kecheng Qian, Lin Gan, Congcong Ma, Xianming Lin

**Affiliations:** 1 The Third Clinical College of Zhejiang University of Traditional Chinese Medicine, Hangzhou, China; 2 Department of Acupuncture, The Third Affiliated Hospital of Zhejiang Chinese Medical University, No. 219 Moganshan Road, Xihu District, Hangzhou, Zhejiang, China, 86 18356130598; 3 Medicine Hangzhou TCM Hospital Affiliated to Zhejiang Chinese Medical University, Hangzhou, China

**Keywords:** ABX, albumin-bound paclitaxel, BBB, blood-brain barrier, glioma, recurrent, single-arm trial, SMES, specific-mode electrical stimulation

## Abstract

**Background:**

Despite advances in surgical resection, radiotherapy, and chemotherapy, the prognosis of recurrent malignant gliomas (rMG) remains poor, with limited efficacy of conventional treatments due to the blood-brain barrier (BBB) hindering drug delivery to the tumor site. Studies have demonstrated that albumin-bound paclitaxel (ABX), while potent in vitro, is restricted in its intravenous use due to BBB limitations. To overcome this, specific-mode electrical stimulation (SMES) has shown promise in transiently opening the BBB, enhancing the accumulation of ABX in glioma tumors. Therefore, this protocol designs a single-center, single-arm, prospective phase II clinical trial aiming to evaluate the safety and clinical efficacy of SMES combined with ABX (SMES+ABX) for treating rMG.

**Objective:**

This study primarily evaluates the safety of SMES+ABX therapy in treating patients with rMG and assesses whether it can improve the 4-month progression-free survival (4m-PFS) rate, while providing data support for future large-scale clinical trials.

**Methods:**

In this study, 20 eligible patients will receive intravenous ABX (135‐175 mg/m²) per 21-day cycle for 6 cycles, combined with SMES for BBB modulation. A Simon 2-stage design will be employed, with the primary end point being the 4m-PFS. Secondary end points include adverse events, disease control rate, objective response rate, duration of disease control, duration of response, Neurological Assessment in Neuro-Oncology score, European Organisation for Research and Treatment of Cancer Quality of Life Questionnaire-Core 30, progression-free survival, and overall survival.

**Results:**

The results will determine the 4m-PFS rate, overall safety profile, secondary efficacy outcomes, and patient-reported quality of life measures. The data will be analyzed upon trial completion. Patient enrollment is scheduled to begin in May 2025. The treatment and primary efficacy assessment phases are anticipated to be completed by January 2027 (allowing for staggered enrollment and a 4-month treatment period for the last enrolled patient). The final survival follow-up for all patients is anticipated to be completed by January 2028 (ie, 1 year after the last patient completes treatment). Data management is currently ongoing, and formal statistical analyses have not yet been performed.

**Conclusions:**

This study aims to evaluate the efficacy and safety of SMES combined with ABX in the treatment of rMG. If successful, the combination could offer a promising therapeutic strategy for this challenging patient population.

## Introduction

Glioblastoma multiforme is the most aggressive primary malignant brain tumor in adults. Even with multimodal treatment, the median overall survival (OS) is only 12‐15 months [1,2]. Nearly all patients experience recurrence, and effective treatment options for recurrence are extremely limited [3,4].

Albumin-bound paclitaxel (ABX), a microtubule-stabilizing agent, exerts antitumor effects by disrupting mitotic spindle dynamics and inducing apoptosis, making it a cornerstone therapy for diverse solid malignancies. In vitro studies have demonstrated the potent antitumor activity of ABX in glioma cell lines (eg, U87 and U251), with mean IC50 values 1400-fold lower than temozolomide [5-8]. However, clinical trials have revealed the limited efficacy of intravenous ABX monotherapy in recurrent glioblastoma [9,10], primarily attributable to insufficient drug accumulation in tumor tissue due to blood-brain barrier (BBB) exclusion [11,12], thereby precluding therapeutic efficacy.

To overcome these limitations, various ABX delivery strategies have been developed, including drug structural modifications and carrier-mediated delivery systems to enhance BBB penetration [13-16]. Recent breakthroughs in focused ultrasound-induced BBB opening have demonstrated 3‐ to 5-fold increases in cerebral ABX concentration in animal models, with significant survival extension. The first human trial (NCT04528680, 2021) preliminarily confirmed the safety of this approach, showing local disease control in 42% of the patients [17,18]. However, despite improved drug delivery to the brain, these strategies face critical challenges, including inconsistent delivery efficiency, safety concerns, and high treatment costs, which limit their clinical translation. Therefore, there is an urgent need for safer, more operable, and cost-effective BBB-penetrating ABX delivery strategies.

Our preliminary studies demonstrated that specific-mode electroacupuncture stimulation (SMES; 2/100 Hz, 3 mA, 6 s on/6 s off, 40 min) effectively opens the BBB, facilitating macromolecule penetration, including Evans blue, fluorescein isothiocyanate–dextran, and neural growth factor [19,20]. The underlying mechanism of SMES in BBB modulation involves the alteration of tight junctions between endothelial cells, transiently disrupting the integrity of the BBB without causing permanent damage or inflammation [21]. This controlled disruption of the BBB effectively enhances the delivery of ABX to glioma tissue, resulting in a significantly higher accumulation at the tumor site than conventional intravenous administration, thereby exerting potent antitumor effects [22].

Although our study demonstrated SMES-mediated macromolecular drug delivery across the BBB, enhancing intracerebral accumulation and therapeutic efficacy, prospective clinical trials are required to validate the clinical benefits of SMES-induced BBB opening for ABX delivery in recurrent malignant gliomas (rMG). Accordingly, we initiated a single-arm, single-center phase II clinical trial to evaluate the safety and efficacy of SMES+ABX therapy for rMG.

## Methods

### Study Design

This was a single-center, single-arm, open-label phase II clinical trial conducted at the Third Affiliated Hospital of Zhejiang Chinese Medical University. The protocol was approved by the Institutional Review Board on January 15, 2025 (approval number: ZSLL-KY-2024-079-01). The study procedure included a brief baseline assessment completed within 48 h after enrollment, an 18-week active treatment phase (a total of 6 cycles), and a subsequent 1-year survival follow-up phase. Patient enrollment is scheduled to begin in May 2025, and the final survival follow-up of all patients is expected to be completed by January 2028. Eligible patients with malignant glioma will receive SMES+ABX therapy, with a 4-month progression-free survival (4m-PFS) rate serving as the primary end point. The secondary end points include adverse events (AEs), disease control rate (DCR), objective response rate (ORR), duration of disease control, duration of response, Neurological Assessment in Neuro-Oncology (NANO) score, European Organisation for Research and Treatment of Cancer Quality of Life Questionnaire-Core 30 (EORTC QLQ-C30), progression-free survival (PFS), and OS. Exploratory biomarker end points include multiomics profiling (transcriptomics, proteomics, and metabolomics) of serial peripheral blood samples collected at baseline and during treatment, aiming to identify potential circulating biomarkers associated with treatment response or resistance mechanisms. The study protocol adheres to the Standards for Reporting Interventions in Clinical Trials of Acupuncture (STRICTA 2010) and SPIRIT (Standard Protocol Items: Recommendations for Interventional Trials) guidelines [23,24].

### Sample Size

The sample size was determined using a Simon 2-stage design, with the primary efficacy end point being the 4m-PFS rate. Previous studies have shown that the 4-month PFS rate for temozolomide in patients with recurrent World Health Organization (WHO) grade 4 glioblastoma does not exceed 25.7% [25,26]. This study set a target threshold of 60% for the 4m-PFS rate based on 3 aspects. First, preliminary exploratory pilot trials conducted by our team suggested activity signals indicating disease stability beyond 4 months in a small number of patients. Second, preclinical studies have confirmed that the combination of SMES and ABX can increase intratumoral drug concentration by approximately 5.5 times and significantly extend animal survival. In addition, focused ultrasound combined with ABX technology, based on a similar principle, achieved a 42% DCR in the first human trial. Together, this evidence supports setting an efficacy goal higher than that of conventional chemotherapy in this study, with the aim of screening treatment strategies with sufficient potential for subsequent confirmatory trials to be conducted. According to calculations using the R software (R Foundation for Statistical Computing; version 4.3.1; 1-sided *α*=.05, power=80%), a total of 20 patients are planned to be enrolled (including an approximate 20% dropout cushion). In the first stage, 9 patients will be enrolled; if fewer than 3 achieve PFS at 4 months, the trial will be terminated early. If the standard is met, 7 additional patients will be recruited in the second stage. If the final overall 4m-PFS rate is less than 56.3% (9/16 patients), the treatment regimen will be considered ineffective.

### Participants and Recruitment Strategy

Patients with rMG will be recruited from the Zhejiang Chinese Medical University, its Third Affiliated Hospital, and other collaborating medical institutions. Recruitment will be primarily conducted through outpatient clinics and inpatient units of the oncology, neurosurgery, and rehabilitation medicine departments. Prior to enrollment, all potential participants will be thoroughly informed of the potential benefits and associated risks related to the exercise intervention component of the study. Written informed consent will be obtained from each participant, who will also be explicitly advised of their right to withdraw from the study at any time without providing a reason for doing so. Upon the completion of the intervention, the participants will be debriefed on the study findings. The research outcomes will be disseminated through publications in peer-reviewed journals and by presenting them at academic conferences.

### rMG Diagnostic Criteria

In this study, postoperative recurrence of malignant glioma was defined as meeting all criteria: (1) WHO grade IV classification per the Chinese Anti-Cancer Association Guidelines for Integrated Diagnosis and Treatment of Glioma (V2.0_2025, dated January 10, 2025) [27]; (2) prior surgical resection; and (3) neuroradiologically confirmed recurrence through cranial magnetic resonance imaging (MRI), which may show either the progression of original lesions or the emergence of new lesions, as adjudicated by board-certified neuro-oncologists. Participant selection will follow the predefined inclusion or exclusion criteria. Textbox 1 details the inclusion, exclusion, withdrawal, dropout, and termination criteria for this study.

Textbox 1. **Inclusion, exclusion, withdrawal, dropout, and termination criteria.**

1. Inclusion criteria

- Histologically confirmed World Health Organization grade IV glioma according to the Chinese Anti-Cancer Association Guidelines for Integrated Diagnosis and Treatment of Glioma (V2.0_2025, dated January 10, 2025)
- Radiologically confirmed disease progression per the RANO 2.0 criteria following surgical resection, with ≥1 measurable contrast-enhancing lesion
- Age 18-70 years (inclusive), any gender
- Dexamethasone dose for mass effect: <6 mg daily (stable for 7 days) or <6 mg average during tapering. Nonmass-effect steroid use was permitted
- Functional status: Karnofsky Performance Status Scale score ≥40
- Adequate organ function (within 14 days): (1) hemoglobin ≥90 g/L; (2) white blood cell ≥3.0×109/L; (3) absolute neutrophil count ≥1500/µL (white blood cell×neutroph); (4) platelets ≥100×109/µL; (5) total bilirubin ≤5×upper limit of normal (ULN); (6) aspartate aminotransferase ≤3×ULN (with bilirubin ≤3×ULN); (7) creatinine ≤1.5 mg/dL or estimated glomerular filtration rate 30-90 mL/min

- Tolerability of electroacupuncture and expected compliance with treatment
- Conscious with preserved:
  - Nociception or discrimination
  - Basic communication capacity

- Willingness to provide written informed consent

1. Exclusion criteria

- Uncontrolled seizure disorder
- Concurrent participation in other interventional trials or within 30 days post participation
- Currently receiving albumin-bound paclitaxel or similar drug treatment
- Severe allergy to albumin-bound paclitaxel or similar compounds
- Pregnant or breastfeeding women
- Diseases affecting cognitive function, such as congenital dementia, alcoholism, drug abuse, or psychotropic substance abuse
- Skin infections at the acupuncture sites
- Patients with conductive foreign objects in their bodies
- Contraindications to gadolinium-enhanced magnetic resonance imaging
- Other acute or chronic diseases, mental illnesses, or abnormal laboratory test values that may increase risks associated with study participation or study drug administration, or interfere with interpretation of study results, and individuals determined by the investigator as ineligible for study participation
- Concurrent other types of antitumor treatments during the trial, such as chemotherapy, radiotherapy, targeted therapy, or immunotherapy

1. Withdrawal, drop-out, and termination criteria

- Individuals who were enrolled but subsequently found not to satisfy the predefined inclusion criteria
- Participants with major protocol deviations related to safety assessments
- Participants who developed serious adverse events or medical complications that necessitated discontinuation of trial participation

### Interventional Methods

**Figure 1.**
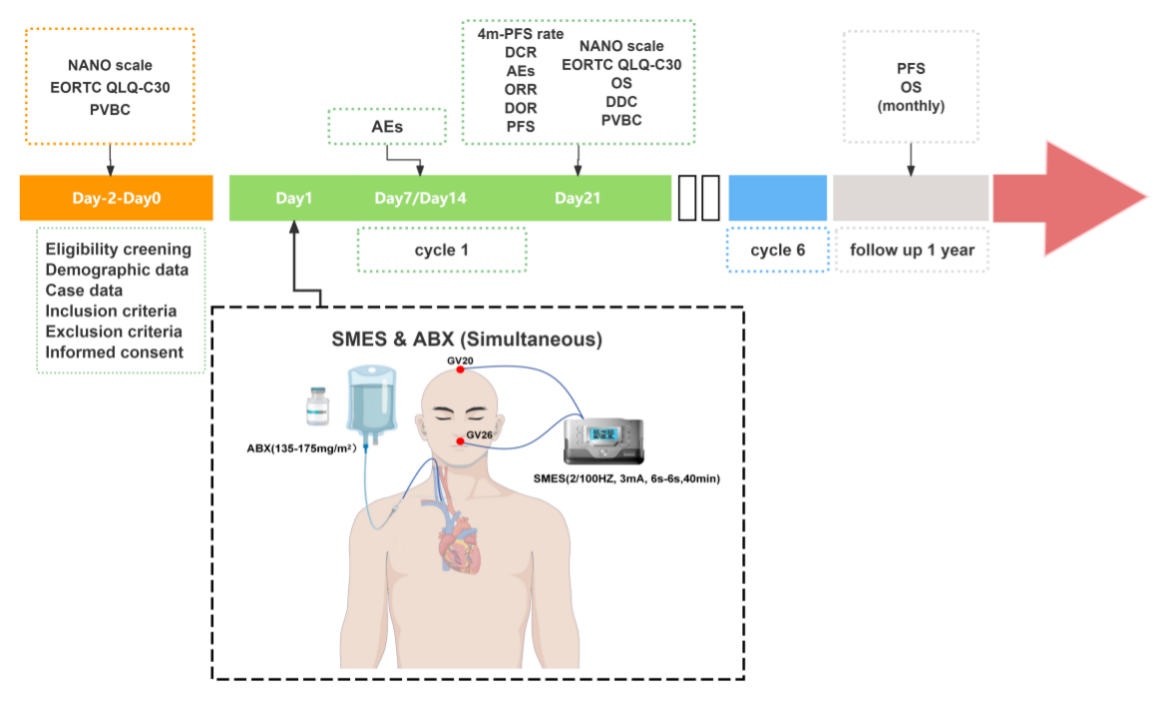
Schematic overview of the experimental protocols. The treatment regimen consisted of 6 cycles, each lasting 21 days, with combined specific-mode electroacupuncture stimulation (SMES) and albumin-bound paclitaxel (ABX) therapy administered on day 1 of each cycle. Adverse events (AEs) were assessed on days 7, 14, and 21 of each cycle. Efficacy was evaluated at the end of each cycle. Postintervention monitoring continued for 1 year, including the monthly follow-up assessments. 4m-PFS: 4-month progression-free survival; DCR: disease control rate; DDC: duration of disease control; DOR: duration of response; EORTC QLQ-C30: European Organisation for Research and Treatment of Cancer Quality of Life Questionnaire-Core 30; NANO: Neurological Assessment in Neuro-Oncology; ORR: objective response rate; OS: overall survival; PVBC: peripheral blood samples collected; PFS: progression-free survival.

Enrolled patients will receive intravenous ABX at a dose of 135‐175 mg/m² on day 1 of each 21-day treatment cycle, with concurrent SMES intervention (Figure 1).

### ABX Treatment Protocol

ABX was administered intravenously at a dose of 175 mg/m² over 40 min. A dose reduction to no less than 135 mg/m² was required in cases of (1) severe neutropenia (absolute neutrophil count <500/mm³ for ≥7 d) or (2) grade ≥3 sensory neuropathy. Treatment was withheld in cases of grade 3 neurotoxicity until resolution to grade ≤2.

### SMES Therapeutic Protocol

The patient was placed in the supine position with full-body relaxation. The skin surrounding GV20 (Baihui) and GV26 (Shuigou) was disinfected. Acupuncture was then administered at DU20, and a 0.25 mm × 40 mm filiform needle was inserted horizontally at a depth of 15‐20 mm. At GV26, a 0.18 mm × 25 mm needle was obliquately inserted toward the nasal septum at a depth of 9‐15 mm. The needles were then connected to an electroacupuncture device (HANS-200; Jisheng Medical Technology). Electrical stimulation was delivered in a dense-disperse wave mode (2/100 Hz) at 1.5‐3.0 mA, which was adjusted to elicit mild muscle twitching near GV26. The treatment lasted 40 minutes, with a 6 seconds on/6 seconds off duty cycle controlled by an external device. The start and end times of SMES device operation were synchronized with the initiation and completion of ABX infusion.

### Assessment and Follow-Up

Tumor response will be evaluated according to the RANO 2.0 criteria [28], incorporating MRI findings and clinical outcomes to determine therapeutic efficacy. Baseline assessments will be conducted within 2 days before treatment initiation, followed by serial evaluations at 3-week intervals, resulting in a total of 7 assessments. AEs will be graded according to CTCAE v5.0. Additionally, the causality between AEs and the investigational drug will be assessed to identify treatment-related AEs. Furthermore, patient-reported outcomes, including quality of life and performance status, will be systematically evaluated.

Post-study follow-up will be conducted for 1 year with monthly telemedicine consultations (via telephone or WeChat) to document survival outcomes. As illustrated in Figure 1, a comprehensive analysis will be performed for each parameter with predefined time frames.

### Concomitant Medications

Concomitant medications for AE management were permitted during the trial, including acid-suppressing agents for gastric protection, hepatoprotective drugs for liver function abnormalities, low-dose corticosteroids for allergy prophylaxis, recombinant human granulocyte colony-stimulating factor (rhG-CSF) or pegylated G-CSF to stimulate leukopoiesis, and recombinant thrombopoietin to stimulate thrombopoiesis. Detailed records were maintained for all concomitant medications, including the generic name, indication, single dose (with units), administration frequency, route, start date, and current status (ongoing or discontinued).

### Rationale for ABX Dose Selection

Although the manufacturer-recommended maximum dose of ABX for breast cancer is 260 mg/m², no clinical guidelines exist for its use in malignant gliomas. Previous studies have demonstrated that ultrasound-mediated BBB disruption significantly enhances ABX delivery in glioma models, achieving 3‐ to 5-fold higher brain concentrations than controls [29]. Similarly, our findings indicated that SMES transiently disrupts the BBB, resulting in a 5.5-fold increase in ABX tumor accumulation relative to controls [22]. Importantly, a phase I clinical trial confirmed the safety of 260 mg/m² ABX when combined with repeated ultrasound-induced BBB opening [17].

However, our preliminary data revealed that SMES combined with ABX doses exceeding 175 mg/m² induced severe dose-limiting toxicities, primarily myelosuppression, resulting in significantly reduced treatment tolerability. Given the discrepancy between preclinical evidence and early clinical safety data, we selected a reduced ABX dose range (135‐175 mg/m²) to ensure patient safety while maintaining the therapeutic feasibility. The dosing strategy was determined by integrating the enhanced drug exposure resulting from BBB disruption technology with a rigorous assessment of clinical tolerability, thereby ensuring scientific validity and clinical relevance.

### Baseline Evaluation and Outcome Measure

Following screening and enrollment, baseline assessments will be conducted within 48 h of enrollment. Comprehensive data collection will include (1) informed consent documentation, (2) medical history, (3) physical examination, (4) laboratory tests, (5) MRI scans (contrast-enhanced brain MRI, magnetic resonance spectroscopy, perfusion-weighted imaging, etc), (6) NANO scale, (7) EORTC QLQ-C30, (8) tumor characteristics (pathological and genomic analyses), (9) detailed demographic information, and (10) peripheral venous blood collection (≈5 mL per cycle) for biobanking and optional future exploratory multiomics studies will be performed. Further specifications are provided in Table 1 and Textbox 2.

**Table 1.** SPIRIT (Standard Protocol Items: Recommendations for Interventional Trials) schedule of enrollment, interventions, and assessments.

Study period	Baseline (day 2 to day 0)	Intervention period (treatment cycles 1 through 6)				Follow-up (1 y)
		Day 1	Day 7	Day 14	Day 21	
Eligibility screening	**✓**					
Demographic data	**✓**					
Case data	**✓**					
Inclusion criteria	**✓**					
Exclusion criteria	**✓**					
Informed consent	**✓**					
SMES+ABX^a^		**✓**				
Outcome assessment						
4m-PFS rate^b^					**✓**	
DCR^c^					**✓**	
AEs^d^			**✓**	**✓**	**✓**	
ORR^e^					**✓**	
NANO^f^ scale	**✓**				**✓**	
EORTC QLQ-C30^g^	**✓**				**✓**	
PFS^h^					**✓**	**✓** ^i^
OS^j^					**✓**	**✓** ^i^
DOR^k^					**✓**	
DDC^l^					**✓**	
PVBC^m^	**✓**				**✓**	

a SMES+ABX: specific-mode electroacupuncture stimulation+albumin-bound paclitaxel.

b 4m-PFS rate: 4-month progression-free survival rate.

c DCR: disease control rate.

d AE: adverse event.

e ORR: objective response rate.

f NANO: Neurological Assessment in Neuro-Oncology.

g EORTC QLQ-C30: European Organisation for Research and Treatment of Cancer Quality of Life Questionnaire-Core 30.

h PFS: progression-free survival.

i Monthly follow-up assessments were conducted during the observation period.

j OS: overall survival.

k DOR: duration of response.

l DDC: duration of disease control.

m PVBC: peripheral venous blood collection.

Textbox 2. **Primary end point and secondary end points.**

Primary end point

- The primary objective of this study is to determine the 4-month progression-free survival rate, defined as the proportion of participants in the full analysis set (FAS) population who remained progression-free beyond 4 months. This end point will be assessed in this study.

Secondary end points

- The disease control rate was defined as the proportion of patients in the FAS population who achieved complete response (CR), progressive disease (PR), or stable disease, as assessed per the RANO 2.0 criteria.
- The objective response rate was calculated as the percentage of FAS patients exhibiting CR or PR, evaluated according to the RANO 2.0 guidelines.
- Overall survival is measured from the initiation of specific-mode electroacupuncture stimulation+albumin-bound paclitaxel (SMES+ABX) therapy until death or censoring at the last follow-up.
- PFS was defined as the time from the first SMES+ABX administration to disease progression (per RANO 2.0), intolerable adverse events, or death, whichever occurred first.
- Duration of response was defined, for patients who achieved an objective response (complete or partial response) to SMES+ABX therapy, as the time from the first documented date of objective response to the date of first documented progressive disease or death from any cause, whichever occurred first.
- Duration of disease control was defined as the time interval from the first radiologically confirmed achievement of disease control (CR, PR, or stable disease) to the first documented progressive disease or death from any cause (whichever occurred first) in patients who received SMES+ABX combination therapy and achieved disease control.
- The NANO scale is a standardized quantitative instrument that evaluates neurofunctional status in patients with neuro-oncology through the systematic assessment of 8 key domains (e.g. motor, sensory, and language functions).
- The European Organisation for Research and Treatment of Cancer Quality of Life Questionnaire-Core 30 questionnaire is a globally validated multidimensional instrument for assessing cancer-related quality of life across 5 functional domains (physical, role, cognitive, emotional, and social) and symptom scales.
- Safety assessments include treatment-emergent adverse events, treatment-related adverse events, and serious adverse events. Treatment-emergent adverse events were defined as adverse events occurring between treatment initiation and study completion. Treatment-related adverse events are adverse events specifically attributable to investigational drugs. Serious adverse events were classified as events resulting in death, life-threatening illness, disability, hospitalization or prolonged hospitalization, or persistent or severe incapacity.

### Analysis Populations

To comprehensively evaluate the efficacy and safety of this single-arm trial and address possible protocol deviations, three analysis populations were predefined in this study. The first is the full analysis set (FAS), in which all patients who signed the informed consent received at least 1 complete cycle of SMES+ABX combination therapy and underwent at least 1 valid posttreatment tumor efficacy assessment. As a single-arm exploratory trial, the FAS is the core population for the primary efficacy analysis in this study (in particular, for decision-making regarding the primary end point of the 4-month PFS rate), aiming to reflect the potential effect of the treatment regimen in eligible patients as fully as possible. The second analysis population is the safety set, in which all patients who signed the informed consent form received at least 1 complete cycle of SMES+ABX combination therapy and underwent at least 1 posttreatment safety assessment. This group will be used for all the safety analyses. The third analysis population is the per protocol set, which is a subset of the FAS, consisting of patients who strictly adhered to the study protocol throughout the treatment period. The per protocol set will serve as a supportive analysis, used to evaluate the treatment effect under ideal compliance, and to compare with FAS results for sensitivity analysis.

### Statistical Analysis Methods

Efficacy assessment will use an analysis framework based on the RANO 2.0 criteria. The primary end point (4m-PFS rate) will be evaluated strictly according to the predefined decision rules of the Simon 2-stage design, with its point estimate and corresponding 95% exact binomial CI (Clopper-Pearson method) calculated. Time-to-event end points (PFS, OS, duration of response, and duration of disease control) will be descriptively analyzed using the Kaplan-Meier method, with median survival times and their 95% CIs reported. An exploratory univariate Cox proportional hazards model was used to assess the association between baseline prognostic factors and survival outcomes. Categorical efficacy end points (such as ORR and DCR) will be reported as frequencies (percentages) with 95% CIs. Continuous variables (such as NANO scores and QLQ-C30 scale scores) will be summarized at each visit using descriptive statistics (mean, standard deviation), and their longitudinal changes will be presented using figures or charts. Given the small sample size, any complex statistical models for analyzing longitudinal changes (such as linear mixed models) will be regarded as exploratory analyses. Safety data will be graded according to the CTCAE v5.0 criteria, and the incidence of AEs and their 95% CIs will be calculated using the Clopper-Pearson method.

Considering that this study is a small-sample, single-arm, exploratory phase II trial, all statistical analyses focused on describing the point and interval estimates for efficacy and safety. Primary conclusions will be drawn based on the decision rules for the primary end point (4m-PFS) outlined in the Simon 2-stage design. All multivariate model analyses and subgroup analyses for secondary end points are exploratory and hypothesis-generating in nature; their results should be interpreted with caution and require validation in future large-scale confirmatory studies. Owing to the limited sample size and low statistical power, the analysis of secondary end points in this study was primarily intended to describe trends and generate hypotheses rather than to conduct confirmatory testing. Statistical analyses will be performed using R 4.3.1 (survival and lme4 packages) and the SAS software (version 9.4; SAS Institute Inc).

### Ethical Considerations

This study was approved by the Institutional Review Board of the Third Affiliated Hospital of Zhejiang Chinese Medical University (approval no. ZSLL-KY-2024-079-01). Before enrollment, potential participants were fully informed of the study protocol, expected benefits, and potential risks using standardized procedures. Separate optional consent was obtained for the collection and long-term storage of peripheral blood samples (≈5 mL per cycle) for future exploratory multiomics research (eg, transcriptomics, proteomics, metabolomics). Participation in this ancillary component is voluntary and does not affect the eligibility for the main trial. Written informed consent was obtained after ensuring a complete understanding of the study. The trial adhered to the principles of the Declaration of Helsinki. All research data were deidentified and stored in encrypted databases to ensure participant confidentiality and data security. The study protocol was registered at ClinicalTrials.gov (Identifier: NCT06818331) and was continuously monitored by an independent data monitoring committee (IDMC). In addition, if there are any changes to the research protocol, we will submit a written application to the research ethics committee, and the committee members will decide whether the protocol needs to be amended.

### Data Management and Monitoring

The research data will be entered into a dedicated database by trained independent personnel according to the study protocol. A multitiered quality control strategy will be implemented to ensure data accuracy, completeness, and consistency of the data. This includes the following: (1) source data verification (100% verification of source documentation for the primary end point [4m-PFS rate]; a minimum of 20% random sampling of secondary end point and safety data for source data verification); (2) medical review (all serious AEs and protocol deviation records will be reviewed by the study physicians); and (3) logical consistency checks (automated verification of data logic and visit timeline adherence using predefined scripts). All data queries will be generated and tracked for resolution using an electronic system. All study-related original data will be stored at the Third Affiliated Hospital of Zhejiang Chinese Medical University.

Any deviations from the approved protocol will be documented in a dedicated protocol deviation log. Deviations will be categorized as minor or major, based on their potential impact on participant safety or data integrity. All major deviations will be promptly reported to the principal investigator and the IDMC.

An independent IDMC will be established to safeguard participant rights, data reliability, and scientific integrity. The committee will consist of independent experts not involved in the trial, including specialists in neuro-oncology, clinical statistics, medical ethics, acupuncture therapeutics, and neuroradiology. The IDMC will review unblinded cumulative safety and efficacy data upon completion of stage 1 of the Simon 2-stage design to assess the risk-benefit profile and provide recommendations for proceeding to stage 2. Following trial completion, the IDMC will review the final data to ensure result integrity. All major protocol deviations and AEs will be reported to the committee, which has the authority to recommend early trial termination based on safety or efficacy concerns. The study will also undergo ongoing oversight and periodic review by the Institutional Review Board of the hospital.

## Results

Patient enrollment is scheduled to begin in May 2025. The treatment and primary efficacy assessment phases are anticipated to be completed by January 2027 (allowing for staggered enrollment and a 4-month treatment period for the last enrolled patient). The final survival follow-up for all patients is anticipated to be completed by January 2028 (ie, 1 year after the last patient completes treatment). Data management is currently ongoing, and formal statistical analyses have not yet been performed.

## Discussion

### Dilemmas and Clinical Needs in the Treatment of rMG

Malignant gliomas, especially the most aggressive form, glioblastoma (WHO grade IV), continue to have an extremely poor prognosis despite the use of standard multimodal treatments, including maximal safe resection, radiotherapy, and temozolomide chemotherapy [30]. The BBB is one of the major factors contributing to unfavorable outcomes, as it severely restricts the delivery of potentially effective chemotherapeutic agents to infiltrating tumor cells [31]. ABX is an effective microtubule stabilizer that has demonstrated efficacy in various solid tumors and exhibits strong cytotoxic effects against glioma cell lines in vitro [32-34]. However, its clinical application in gliomas has been hampered by insufficient accumulation in the brain following systemic administration [14,15]. Current research shows that 3 strategies—nanoformulation engineering, coadministration with BBB modulators, and physical or chemical disruption of the BBB—can enhance the ability of drugs to cross the BBB [35-37]. Nevertheless, there are many challenges in clinical translation owing to issues such as technical complexity, high costs, and safety concerns. Thus, developing strategies that can open the BBB in a brief, safe, and controllable manner is a key challenge in neuro-oncology. The SMES combined with ABX therapy explored in this study aims to provide a novel, noninvasive, cost-effective, and easy-to-operate solution to address this challenge.

### Innovation and Design Rationality

This study innovatively applies a physical BBB modulation technology, SMES, in a prospective manner to the chemotherapy treatment of patients with rMG. The theoretical foundation is based on our previous series of studies, which demonstrated that electroacupuncture stimulation with specific parameters (2/100 Hz, 3 mA, 6 s on/6 s off) can safely increase BBB permeability by transiently and reversibly regulating tight junction proteins (such as ZO-1 and Occludin), thereby promoting the delivery of macromolecules (such as Evans blue and fluorescein isothiocyanate–dextran), and even therapeutic proteins (such as nerve growth factor) into the brain [19,20]. More importantly, in rat models of glioma, SMES significantly increased the accumulation of ABX in tumor tissue (by approximately 5.5 times) and enhanced its antitumor efficacy [22]. These studies provide robust preclinical evidence for translational research.

In terms of study design, we adopted a single-center, single-arm, Simon 2-stage phase II clinical trial, which is a rational choice for evaluating the preliminary efficacy and safety of an innovative therapy. The primary end point was set as 4m-PFS, aligning with conventions in clinical trials of recurrent high-grade gliomas and enabling a rapid and effective assessment of whether the treatment brings about clinically meaningful disease control [25,26]. The secondary end points encompass multiple dimensions, including efficacy (such as ORR, DCR, and OS), neurological function (NANO scale), patient-reported quality of life (EORTC QLQ-C30), and safety, aiming to comprehensively evaluate the SMES+ABX therapy. The chosen ABX dose (135‐175 mg/m²) was determined based on mechanistic, safety, and clinical practice considerations. The upper limit (175 mg/m²) was set as the starting dose based on dose-limiting toxicity (mainly grade 3‐4 myelosuppression) observed in our previous studies, ensuring a safe initiation. The lower limit (135 mg/m²) was established considering the following: preclinical studies showed that SMES could increase ABX concentration in tumors by approximately 5.5 times [22]; from a drug exposure perspective, even with a reduced systemic dose, enhanced delivery via SMES could still achieve effective local concentrations; published clinical trials combining focused ultrasound BBB opening with ABX have confirmed the safety of a 260 mg/m² dose [17], so 135 mg/m² ensures a sufficient safety margin; and this dose also conforms to the standard oncology dose reduction (approximately 25%), which facilitates clinical management while retaining clear pharmacological activity after reduction. This dosing range reflects a cautious balance between therapeutic potential and patient safety under the SMES-enhanced delivery mechanism.

### Explanation of Expected Outcomes and Potential Mechanisms

If this study meets its predefined primary end point (ie, ≥9 out of 16 patients achieve 4m-PFS, with an overall response rate of ≥56.3%), it will strongly suggest that the SMES combined with the ABX regimen has potential advantages in controlling the progression of rMG. The reason for such positive outcomes may lie in the SMES, through its specific electrophysiological stimulation, acting on the trigeminal nerve-cerebrovascular system, resulting in local cerebral vasodilation and hemodynamic changes, which in turn lead to a transient and reversible structural remodeling of the tight junctions between endothelial cells [21,38-43]. This change in BBB permeability allows more ABX to cross the barrier, achieving effective concentrations at the tumor site, thereby exerting strong cytotoxic effects.

Regarding the secondary end points, the regulatory effect of SMES on local cerebral hemodynamics [39-43], combined with the increased concentration of ABX at the tumor site [22], may allow the patients’ neurological status (NANO score) to remain stable or even improve. Moreover, the maintenance or improvement of neurological function, together with symptom alleviation brought about by disease control, may be reflected in stable or improved quality of life scores (EORTC QLQ-C30). Safety was the top priority in this study. As a modified electroacupuncture therapy, SMES should have a favorable safety profile; AEs are still expected to be mainly due to the known toxicities of ABX (such as sensory neuropathy and myelosuppression). However, it is essential to closely monitor whether increased ABX concentrations in the brain induced by SMES affect central nervous system toxicity.

### Limitations

This study had several inherent design limitations. First, the single-arm design lacks concurrent randomized controls; therefore, efficacy assessments rely on historical controls, which may introduce selection bias and be influenced by advances in treatment over time, thus limiting the certainty of the conclusions. Second, although the Simon 2-stage design was used to improve exploratory efficiency, the limited sample size (planned for 16 cases, with up to 20 cases to account for dropouts) may result in insufficient statistical power for secondary end points and make it difficult to identify rare AEs or differences in efficacy among the subgroups. Third, patients with rMG exhibit heterogeneity in molecular pathology and treatment history, which may potentially affect their treatment response. Fourth, the planned multiomics analyses of serial peripheral blood samples are exploratory and hypothesis-generating; their technical feasibility, biological interpretability, and clinical relevance remain to be determined, and any findings will require independent validation in larger cohorts. However, phase II clinical trials are essentially exploratory rather than confirmatory in nature; this study aims to provide preliminary evidence for the potential benefits of the SMES combined with ABX regimen. If the results are positive, a phase III randomized controlled trial will be required to further validate its efficacy.

### Conclusion

This study adopted the Simon 2-stage design to conduct a single-arm phase II clinical trial enrolling 16 patients, aiming to evaluate the efficacy and safety of SMES combined with ABX in the treatment of rMG. The primary end point of the study is 4m-PFS, while the secondary end points include ORR, DCR, OS, NANO score, and EORTC QLQ-C30. Safety assessments include treatment-related AEs and serious AEs. If successful, this study will provide an innovative, noninvasive, and cost-effective treatment strategy for this disease, which has an extremely poor prognosis, and will lay a foundation for subsequent studies.

## Supplementary material

10.2196/84593 Checklist 1 SPIRIT checklist for protocol of a clinical trial.

## Abbreviations

4m-PFS: 4-month progression-free survival
ABX: albumin-bound paclitaxel
AE: adverse event
BBB: blood-brain barrier
DCR: disease control rate
EORTC QLQ-C30: European Organisation for Research and Treatment of Cancer Quality of Life Questionnaire-Core 30
FAS: full analysis set
IDMC: independent data monitoring committee
MRI: magnetic resonance imaging
NANO: Neurological Assessment in Neuro-Oncology
ORR: objective response rate
OS: overall survival
PFS: progression-free survival
rMG: recurrent malignant gliomas
SMES: specific-mode electroacupuncture stimulation
SPIRIT: Standard Protocol Items: Recommendations for Interventional Trials
WHO: World Health Organization
